# Chronographic Imprint of Age-Induced Alterations in Heart Rate Dynamical Organization

**DOI:** 10.3389/fphys.2015.00201

**Published:** 2015-07-14

**Authors:** Danuta Makowiec, Dorota Wejer, Agnieszka Kaczkowska, Marta Żarczyńska-Buchowiecka, Zbigniew R. Struzik

**Affiliations:** ^1^Institute of Theoretical Physics and Astrophysics, University of Gdańsk, Gdańsk, Poland; ^2^Faculty of Applied Physics and Mathematics, Gdańsk University of Technology, Gdańsk, Poland; ^3^1st Chair & Clinic of Cardiology, Medical University of Gdańsk, Gdańsk, Poland; ^4^RIKEN Brain Science Institute, Wako-shi, Japan; ^5^Graduate School of Education, The University of Tokyo, Tokyo, Japan

**Keywords:** short-term heart rate variability, aging, deceleration capacity, entropy rate

## Abstract

Beat-to-beat changes in the heart period are transformed into a network of increments between subsequent RR-intervals, which enables graphical descriptions of short-term heart period variability. Three types of such descriptions are considered: (1) network graphs arising from a set of vertices and directed edges, (2) contour plots of adjacency matrices A, representing the networks and transition matrices T, resulting from A, and (3) vector plots of gradients of the matrices A and T. Two indices are considered which summarize properties of A and T: the approximate deceleration capacity and the entropy rate. The method, applied to time series of nocturnal RR-intervals recorded from healthy subjects of different ages, reveals important aspect of changes in the autonomic activity caused by biological aging. Independent of the subject’s age, following accelerations, a pendulum-like dynamics appears. With decelerations, this dynamics develops in line with the subject’s age. This aging transition can be graphically visualized by vectors connecting the maxima of the transition probabilities of T, which, metaphorically, resemble a chronometer or the hands of a clock.

## 1 Introduction

The two branches of the autonomic nervous system (ANS), the sympathetic and vagal subsystems, cooperate to maintain homeostasis in the cardiovascular (CV) system, namely the continuous controlled movement of blood through the whole organism to transport nutrients to organ tissues – to every cell of the organism (Guyton and Hall, 2006). With advancing age, a gradual impairment in the functioning of the complex interplay between these two subsystems develops (Esler et al., 1995). The phenomenon of such age-related ANS-CV deterioration is revealed as noticeable alterations in the cardiac interbeat RR-interval dynamics. Signals with RR-intervals derived from long-term ECG recordings have been widely used in assessing ANS responses during normal activities in health and disease (Reardon and Malik, 1996; Pikkujämsä et al., 1999; Crasset et al., 2001; Struzik et al., 2004, 2006; Shimazu et al., 2005; Beckers et al., 2006; Meersman and Stein, 2007; Monahan, 2007; Stein et al., 2009; Callegaro and Taylor, 2010; Makowiec et al., 2011). The reasons for the observed variations are not yet completely understood (Goldberger et al., 2008). Nevertheless, heart rate variability is a recognized surrogate index for cardiac autonomic function of the sinus node and ventricles, and is a marker of im-/balance in the sympathetic and vagal activities (Goldberger et al., 2008; Poirier, 2014).

In particular, in Reardon and Malik (1996), a significant decrease with age was shown in indices of heart rate variability (HRV) based on an overall HRV estimation. But for indices which describe short-term components of HRV, and are therefore expected specifically to reflect vagal modulation, no significant change was observed. Struzik et al. (2006) reported a progressive alteration in the sympathetic/parasympathetic balance and put forward the “autonomic aging” hypothesis, involving emerging parasympathetic dominance. Alteration in the long-range organization, and a loss of complexity have been reported by Pikkujämsä et al. (1999), Beckers et al. (2006), and Viola et al. (2011), though fractal scale-invariance and non-linear properties have been claimed to remain stable with the advance of age (Schmitt and Ivanov, 2007). Schmitt et al. (2009) further argued that a sleep phase-based, well-pronounced cardiac risk stratification pattern remains unchanged with age, even in elderly patients. Yet the elementary linear measures of autonomic activity are known to be strongly influenced by both the sleep phase structure and aging (Brandenberger et al., 2003) – apparently through altered respiratory patterns in the elderly.

The ECG data are of fixed resolution, which leads to the natural discretization of values for the RR-intervals. As a consequence, the values of changes in the RR-intervals: accelerations and decelerations also become multiples of the intrinsic signal resolution. Such quantification has an effect in setting up a state space of a finite size, the elements of which can consistently be labeled by values representing the RR-increments. As a consequence, a framework can be obtained, which is particularly suitable for analysis by methods based on the symbolic dynamics approach, including network representation (Donner et al., 2010; Campanharo et al., 2011). For example, one can ask whether the subsequent change in the RR-interval is related to the current change or not, and if so, what this relation looks like. Different mechanisms within the heart rate regulatory system are assumed to be responsible for accelerations and for decelerations (Guyton and Hall, 2006). Adjustments caused by the sympathetic system are slow, on a scale of seconds, whereas adjustments caused by the vagal system are fast (Stein and Pu, 2012; Poirier, 2014). Therefore, qualification and quantification of changes in RR-intervals provide insights into mechanisms driving the heartbeat dynamics.

In the following, we describe a geometrically inspired method, which enables the assessment of the dependencies between subsequent changes in the RR-intervals from two aspects: the visual aspect, due to which an intuitive description is given, and the quantitative aspect, which provides the rationale behind our argumentation. In particular, we apply the method to analyze beat-to-beat variability of nocturnal heart periods of healthy people of different ages, from young people at the age of twenty to the elderly at ages of up to ninety. Two indices are considered to draw conclusions about the geometric properties observed. The entropy rate gives an overall measure of changes in the heard period resulting from the Markov chain representation. The deceleration capacity (Schumann et al., 2010) is tuned to be concentrated on properties of large decelerations, hence aims to quantify events in which the vagal activity is expected to be especially high.

This work is a continuation of our earlier investigations into the usability of tools from network theory in the assessment of RR-time interval signals (Makowiec et al., 2014, 2015a,b).

## 2 Materials and Methods

### 2.1 Signal preprocessing

Twenty-four-hour Holter ECG recordings during a normal sleep–wake rhythm were obtained from healthy volunteers without any known cardiac history. Individuals with a high risk of obstructive sleep apnea [see Table 2 in Epstein et al. (2009)] were not included in the study. All the participants in the study gave their informed consent. The study complied with the Declaration of Helsinki and was approved by the Bioethics Committee of the Medical University of Gdańsk.

The participants, in total 194 people, were pooled into age-dependent groups: *twenties*: 36 subjects (18 women, 18 men, age: 20–29), *thirties*: 26 subjects (13 women, 13 men, age: 30–39), *forties*: 36 subjects (16 women, 20 men, age: 40–49), *fifties*: 32 subjects (13 women, 19 men, age: 50–59), *sixties*: 24 subjects (11 women, 13 men, age: 60–69), *seventies*: 22 subjects (10 women, 12 men, age: 70–79), and *eighties*: 18 subjects (11 women, 7 men, age: 80–89). Holter recordings were first analyzed using Del Mar Reynolds Impresario software and screened for premature, supraventricular and ventricular beats, missed beats, and pauses. Then, the signals were thoroughly corrected manually and annotated correspondingly. The sleep hours were selected for each signal individually, according to the day–night transition observed in the length of the RR-intervals. Finally, a six-h period, covering the longest RR-intervals, was extracted. Perturbations in a signal (artifacts or any but normal-to-normal RR-intervals), consisting of less than five consecutive RR-intervals, were replaced by the medians calculated from the last seven normal RR-intervals. Other perturbations were deleted. Ultimately, the signals studied were constructed from at least 20,000 normal-to-normal RR-intervals. The Holter equipment used by us provided signals with 128 Hz sampling frequency, which sets the resolution of the RR-intervals to approximately 8 ms. As a consequence, all the values obtained for RR-intervals, and in turn for RR-increments, are multiples of 8 ms.

If **RR** = {*RR*_0_, … , *RR_i_*, … , *RR_N_*} is the time sequence of RR-intervals, *i* is the time index, then the signal of RR-increments is defined as **ΔRR** = {*δRR*_1_ ,… , δ*RR_i_*, … , δ*RR_N_*} with *δRR_i_* = *RR_i_* − *RR_i−1_*. Hence, an event described by *δRR_i_* > 0 denotes a deceleration and each event characterized by *δRR_i_* < 0 is an acceleration. When *δRR_i_* = 0, we say a no-change event has taken place.

The state space of the signal values consists of a finite number of multiplies of 8 ms, namely 0, ±8, ±16, … , ms which, when arranged from the smallest acceleration to the largest deceleration, are referred as the following set:

(1) $$\Delta_{J}\in \left\{-\Delta_{K},\ldots ,0,\ldots ,\Delta_{K}\right\},\;\Delta_{K}=\underset{i}{\max}\;\left\{\left|\delta RR_{i}\right|\right\}.$$

By applying binning to RR-intervals, e.g., with a bin equal to 48 ms, the corresponding set of state space values (1) decreases, which allows for a more compact presentation of the results. Unless otherwise described, the results presented were obtained with the accuracy of the intrinsic signal resolution.

### 2.2 Assessment of RR-increments

In a sequence of pairs {(*δRR_i_*, *δRR_i_*_+1_), *i * = 1, …, *N*}, constructed from a given signal **ΔRR**, we search for all pairs (Δ*_I_*, Δ*_J_*), *I*, *J * = − *K*, …, *K*, and then normalize the count of each pair to obtain the (*I*, *J*)-th element of a square matrix **A**:

(2) $$A_{\mathrm{IJ}}=\frac{\left|\left\{\left(\delta RR_{i},\delta RR_{i+1}\right):\delta RR_{i}=\Delta_{I},\delta RR_{i+1}=\Delta_{J}\right\}\right|}{N}.$$

In this way, the matrix **A** contains probabilities that events (Δ*_I_*, Δ*_J_*) occur one by one in a time sequence. Matrix **A** can be read as an adjacency matrix of a directed and weighted network with vertices defined by (1) and with directed edges leading from Δ*_I_* to Δ*_J_*, the weight of which is given by *A_IJ_*.

Straightforward algebra ensures that:

(3) $$\begin{array}{ll} \frac{1}{4}\left(RR_{i}+RR_{i+1}-RR_{i-1}-RR_{i-2}\right) & =\frac{1}{4}\left(\delta RR_{i-1}+\delta RR_{i}+\delta RR_{i}+\delta RR_{i+1}\right), \end{array}$$

which means that the deceleration capacity (DC) obtained by the phase-rectified signal averaging method (PRSA) (Schumann et al., 2010) can be considered to be a function in the space of RR-increments. Moreover, we can use the general formula of DC for decelerations of only a given size, and in this way obtain the index of DC limited to the given decelerations. In particular, for some presumed deceleration size limit Δ*_D_*, information about decelerations and accelerations from the matrix **A** can be used to estimate the approximate DC based on the formula:

(4) $$DC_{Α}=\frac{1}{4}\;\left[\sum_{{\text{Δ}}_{I}\geq {\text{Δ}}_{D}}\sum_{{\text{Δ}}_{K}}({\text{Δ}}_{K}+{\text{Δ}}_{I})\;A_{KI}\;+\sum_{{\text{Δ}}_{I}\geq {\text{Δ}}_{D}}\sum_{{\text{Δ}}_{J}}({\text{Δ}}_{I}+{\text{Δ}}_{J})\;A_{IJ}\right]$$

In our further calculations, we assume Δ*_D_ * = 40 ms, because this value corresponds to a 5% change in the length of RR-intervals.

For any adjacency matrix **A**, a corresponding transition matrix **T** can be introduced as follows:

(5) $$T_{IJ}=\frac{A_{IJ}}{\sum {\;}_{J}\;A_{IJ}}=P\left({\text{Δ}}_{J}|{\text{Δ}}_{I}\right).$$

Matrix **T** describes the conditional probability of observing Δ*_J_* if an increment Δ*_I_* has taken place. Similarly to matrix **A**, matrix **T** can also be represented as a directed and weighted network with the same vertices as for matrix **A**, but with edges corresponding to the probabilities of transitions from a given vertex.

Transition matrix **T** captures and represents a Markov process, which underlies changes in the system studied. Matrix **T** is right stochastic, and consequently its stationary state can be inferred. This stationary state μ comprises the eigenvector of **T** corresponding to eigenvalue 1. Consequently, we can calculate the entropy rate as follows:

(6) $$S_{T}=-\sum_{I=-K}^{K}\mu_{I}\sum_{J=-K}^{K}T_{IJ}\text{ ln }T_{IJ}.$$

Finally, for each matrix **A** and **T**, we can construct gradient matrices, **GA** and **GT**, respectively, consisting of vectors which describe the direction and power of local changes:

(7) $$\begin{array}{cc} GA_{\mathrm{IJ}} & =\left(A_{I,J+1}-A_{I,J-1},A_{I+1,J}-A_{I-1,J}\right)\\ GT_{\mathrm{IJ}} & =\left(T_{I,J+1}-T_{I,J-1},T_{I+1,J}-T_{I-1,J}\right). \end{array}$$

## 3 Results

**Figure 1:**
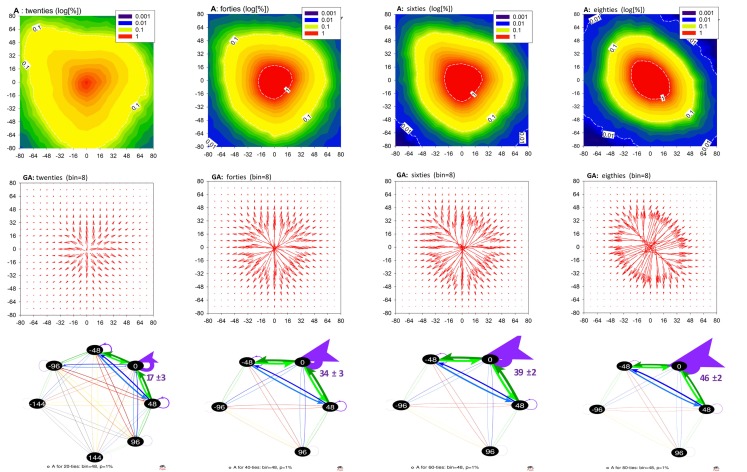
**Networks of transitions for aging population**. **Top row**: matrices A averaged from matrices obtained from RR-signals pooled according to the age groups. **Middle row**: gradient matrices **GA** calculated from mean **A**. **Bottom row**: networks resulting from matrices **A** when RR-signals were binned with *bin * = 48 ms. Transitions are shown only between vertices of which the probability of occurrence is >1%. The growing role of small increments, as age progresses, is illustrated by providing numbers for the no-change events. Network graphs are visualized by Pajek software (Batagelj and Mrvar, 2002).

**Figure 2:**
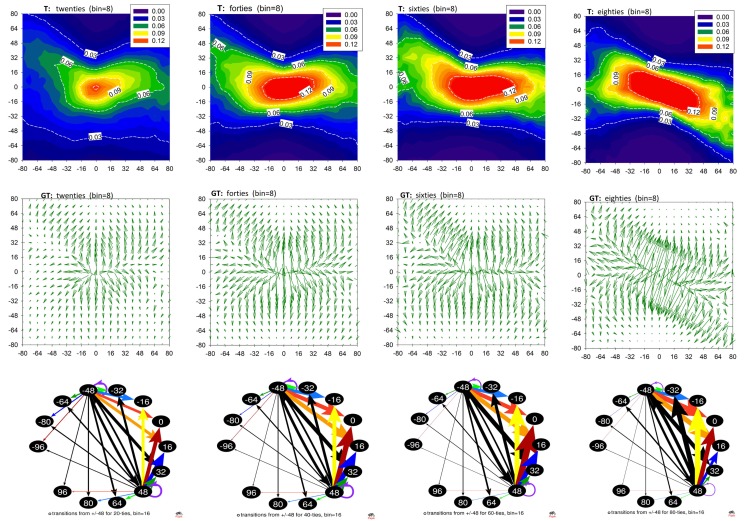
**Transition matrices in aging population**. **Top row**: matrices T averaged from matrices obtained from RR-signals pooled according to the age groups. **Middle row**: gradient matrices **GT** calculated from mean **T**. **Bottom row**: networks resulting from matrices **T** when RR-signals were binned with *bin * = 16 ms. Transitions are shown for vertices +48 and −48 to visualize changes caused by the progression of age. Network graphs are visualized by Pajek software (Batagelj and Mrvar, 2002).

In Figures 1 and 2, and Table 1, we show the results of the method applied to the data described. These plots show properties of the mean matrices **A** and **T** obtained by averaging matrices calculated for individual subjects and then pooled into the age groups. Unfortunately, graphs of networks constructed at the signals’ resolution accuracy, namely 8 ms, would be barely legible. Therefore, these networks are presented as contour plots of the adjacency and transition matrices; see the first rows of Figures 1 and 2. The plots of **A** describe the probability distributions of observing in a sequence Δ*_I_* – shown on the horizontal axis, and Δ*_J_* – shown on the vertical axis. The plots of **T** show the conditional probability of observing Δ*_J_* – shown on the vertical axis, conditioned by Δ*_I_* – shown on the horizontal axis. The presentation of accelerations and decelerations is restricted to events smaller than 80 ms. However, such an interval of increments covers the crucial aspects of the heart beat dynamics.

**Table 1:** **Core parts of T: *P*(Δ_*J*_|Δ_*I*_) in the case of *bin* = 16 ms for different age groups are given as the group mean ± SEM**.

Δ_*J*_ →Δ_*I*_ ↓	−80	−64	−48	−32	−16	0	16	32	48	64	80
**TWENTIES**											
−80	3 ± 0.4	4 ± 0.5	5 ± 0.5	6 ± 0.6	7 ± 0.6	8 ± 0.7	9 ± 0.7	10 ± 0.9	9 ± 1.0	8 ± 1.0	6 ± 0.9
−64	3 ± 0.4	4 ± 0.5	6 ± 0.5	7 ± 0.5	8 ± 0.6	9 ± 0.7	10 ± 0.8	11 ± 1.0	10 ± 1.0	8 ± 0.9	5 ± 0.8
−48	4 ± 0.4	5 ± 0.4	6 ± 0.5	8 ± 0.6	10 ± 0.7	10 ± 0.7	**11** ± **1.0**	**11** ± **1.0**	**9** ± **0.9**	6 ± 0.6	4 ± 0.5
−32	4 ± 0.4	5 ± 0.4	7 ± 0.5	9 ± 0.7	11 ± 1.0	13 ± 1.2	13 ± 1.4	11 ± 1.0	7 ± 0.6	5 ± 0.4	3 ± 0.3
−16	3 ± 0.3	5 ± 0.4	7 ± 0.6	9 ± 0.8	14 ± 1.6	18 ± 2.8	15 ± 2.4	9 ± 0.9	5 ± 0.3	3 ± 0.2	2 ± 0.2
0	3 ± 0.3	5 ± 0.4	7 ± 0.6	10 ± 1.0	17 ± 2.7	22 ± 3.8	14 ± 2.1	7 ± 0.6	4 ± 0.3	2 ± 0.1	1 ± 0.1
16	3 ± 0.3	5 ± 0.4	7 ± 0.6	10 ± 0.9	15 ± 1.9	19 ± 3.0	15 ± 1.8	9 ± 0.8	5 ± 0.3	3 ± 0.2	2 ± 0.1
32	4 ± 0.3	5 ± 0.5	7 ± 0.6	9 ± 0.7	12 ± 1.0	16 ± 1.6	15 ± 1.6	10 ± 1.0	6 ± 0.5	3 ± 0.3	2 ± 0.2
48	4 ± 0.4	5 ± 0.5	7 ± 0.6	8 ± 0.7	11 ± 0.7	14 ± 1.1	**14** ± **1.4**	**10** ± **0.9**	**7** ± **0.6**	4 ± 0.3	3 ± 0.3
64	4 ± 0.5	5 ± 0.6	7 ± 0.7	9 ± 0.9	10 ± 0.9	13 ± 1.1	12 ± 1.0	9 ± 0.8	7 ± 0.6	4 ± 0.4	3 ± 0.3
80	4 ± 0.5	6 ± 0.7	7 ± 0.9	9 ± 1.0	11 ± 1.2	11 ± 1.2	10 ± 0.9	8 ± 0.7	6 ± 0.5	4 ± 0.4	3 ± 0.3
**FORTIES**											
−80	2 ± 0.7	3 ± 0.7	5 ± 0.8	6 ± 0.8	8 ± 1.0	10 ± 1.4	11 ± 2.0	12 ± 2.5	13 ± 3.1	10 ± 2.7	7 ± 2.0
−64	2 ± 0.5	3 ± 0.6	5 ± 0.6	7 ± 0.8	10 ± 0.9	12 ± 1.2	13 ± 1.6	13 ± 2.1	12 ± 2.2	8 ± 1.9	5 ± 1.2
−48	2 ± 0.4	3 ± 0.5	5 ± 0.6	9 ± 0.7	13 ± 0.9	16 ± 1.1	**16** ± **1.3**	**14** ± **1.7**	**10** ± **1.5**	5 ± 1.0	3 ± 0.6
−32	1 ± 0.3	3 ± 0.3	5 ± 0.4	10 ± 0.7	17 ± 1.2	21 ± 1.3	18 ± 1.2	13 ± 1.0	6 ± 0.7	3 ± 0.4	1 ± 0.2
−16	1 ± 0.2	2 ± 0.3	4 ± 0.4	9 ± 0.6	20 ± 1.5	27 ± 2.6	19 ± 1.6	9 ± 0.6	4 ± 0.3	1 ± 0.2	1 ± 0.1
0	1 ± 0.2	2 ± 0.3	4 ± 0.4	9 ± 0.6	21 ± 1.9	32 ± 4.2	18 ± 1.6	7 ± 0.4	2 ± 0.2	1 ± 0.1	0 ± 0.1
16	1 ± 0.2	2 ± 0.3	5 ± 0.4	10 ± 0.6	20 ± 1.4	28 ± 2.4	19 ± 1.4	8 ± 0.5	3 ± 0.2	1 ± 0.1	1 ± 0.1
32	2 ± 0.4	3 ± 0.5	5 ± 0.6	9 ± 0.7	16 ± 0.9	23 ± 1.6	19 ± 1.5	10 ± 0.6	4 ± 0.4	2 ± 0.2	1 ± 0.1
48	2 ± 0.6	4 ± 0.7	6 ± 1.0	9 ± 1.1	14 ± 1.2	19 ± 1.6	**17** ± **1.8**	**11** ± **1.0**	**6** ± **0.7**	3 ± 0.4	2 ± 0.3
64	3 ± 0.7	5 ± 1.0	7 ± 1.2	10 ± 1.5	12 ± 1.7	16 ± 2.1	14 ± 2.0	11 ± 1.6	7 ± 1.2	4 ± 0.8	2 ± 0.5
80	4 ± 0.9	5 ± 1.1	7 ± 1.5	9 ± 1.8	11 ± 2.0	13 ± 2.4	12 ± 2.3	10 ± 2.1	7 ± 1.4	5 ± 1.1	3 ± 0.7
**SIXTIES**											
−80	1 ± 0.5	3 ± 1.0	5 ± 1.7	7 ± 2.0	9 ± 2.2	11 ± 2.5	12 ± 3.2	13 ± 3.5	13 ± 3.4	10 ± 3.1	7 ± 2.0
−64	1 ± 0.4	2 ± 0.7	4 ± 1.0	7 ± 1.5	10 ± 1.7	13 ± 2.2	14 ± 2.7	16 ± 3.1	13 ± 3.1	9 ± 2.5	5 ± 1.5
−48	1 ± 0.3	2 ± 0.5	4 ± 0.8	7 ± 1.1	12 ± 1.4	16 ± 1.3	**17** ± **1.6**	**17** ± **2.4**	**12** ± **2.3**	6 ± 1.3	3 ± 0.7
−32	1 ± 0.2	2 ± 0.4	3 ± 0.5	8 ± 0.8	15 ± 1.1	22 ± 1.3	21 ± 1.4	16 ± 1.7	7 ± 1.0	3 ± 0.5	1 ± 0.3
−16	1 ± 0.2	1 ± 0.3	3 ± 0.4	9 ± 0.6	19 ± 1.2	28 ± 2.6	22 ± 1.7	11 ± 0.7	4 ± 0.3	1 ± 0.2	1 ± 0.1
0	1 ± 0.1	1 ± 0.3	3 ± 0.4	10 ± 0.6	22 ± 1.9	33 ± 4.4	19 ± 1.7	7 ± 0.4	2 ± 0.2	1 ± 0.1	0 ± 0.1
16	1 ± 0.2	2 ± 0.3	4 ± 0.5	11 ± 0.7	22 ± 1.5	31 ± 2.6	18 ± 1.2	7 ± 0.5	2 ± 0.2	1 ± 0.1	0 ± 0.1
32	1 ± 0.3	2 ± 0.5	5 ± 0.8	11 ± 1.2	20 ± 1.3	27 ± 1.9	19 ± 1.4	8 ± 0.8	3 ± 0.5	1 ± 0.2	1 ± 0.1
48	2 ± 0.5	3 ± 0.8	**6** ± **1.0**	**11** ± **1.7**	**18** ± **1.9**	23 ± 2.6	**17** ± **2.1**	**9** ± **1.3**	**5** ± **0.8**	2 ± 0.4	1 ± 0.2
64	2 ± 0.8	4 ± 1.0	7 ± 1.4	12 ± 2.1	17 ± 2.8	19 ± 3.5	15 ± 3.3	9 ± 1.9	5 ± 1.2	3 ± 0.8	1 ± 0.5
80	4 ± 1.2	5 ± 1.4	8 ± 2.0	13 ± 2.9	14 ± 3.2	16 ± 4.3	14 ± 3.8	8 ± 1.9	6 ± 1.6	3 ± 1.0	2 ± 0.5
**EIGHTIES**											
−80	2 ± 1.1	3 ± 1.5	4 ± 2.3	8 ± 2.7	10 ± 3.1	12 ± 3.5	11 ± 2.8	9 ± 2.4	9 ± 2.4	6 ± 1.8	3 ± 1.1
−64	1 ± 0.8	2 ± 1.0	4 ± 1.8	7 ± 2.3	11 ± 2.9	16 ± 3.8	14 ± 3.1	13 ± 3.1	10 ± 2.3	6 ± 1.2	3 ± 0.8
−48	1 ± 0.3	2 ± 0.7	3 ± 0.9	7 ± 1.7	13 ± 2.5	20 ± 3.5	**20** ± **2.9**	**16** ± **2.4**	**9** ± **1.6**	4 ± 0.8	2 ± 0.5
−32	0 ± 0.1	1 ± 0.3	2 ± 0.5	5 ± 0.9	14 ± 1.7	24 ± 1.9	26 ± 1.7	16 ± 1.6	6 ± 0.9	2 ± 0.4	1 ± 0.2
−16	0 ± 0.1	0 ± 0.1	1 ± 0.2	5 ± 0.5	16 ± 1.1	33 ± 2.7	29 ± 2.5	11 ± 0.7	3 ± 0.4	1 ± 0.2	0 ± 0.1
0	0 ± 0.1	0 ± 0.1	1 ± 0.2	6 ± 0.5	22 ± 1.7	40 ± 4.7	21 ± 1.7	6 ± 0.4	2 ± 0.3	1 ± 0.1	0 ± 0.0
16	0 ± 0.1	1 ± 0.2	3 ± 0.4	10 ± 0.7	30 ± 2.7	33 ± 2.7	15 ± 1.3	5 ± 0.6	1 ± 0.3	0 ± 0.1	0 ± 0.1
32	1 ± 0.3	2 ± 0.5	6 ± 0.9	18 ± 1.9	28 ± 1.8	24 ± 2.0	12 ± 1.6	5 ± 0.9	2 ± 0.5	1 ± 0.3	0 ± 0.2
48	2 ± 0.7	5 ± 1.2	**12** ± **2.6**	**19** ± **2.9**	**22** ± **3.0**	17 ± 2.8	10 ± 2.1	5 ± 1.3	2 ± 0.8	1 ± 0.5	1 ± 0.4
64	5 ± 1.1	10 ± 2.2	15 ± 3.3	16 ± 3.1	15 ± 2.7	13 ± 2.8	8 ± 2.3	4 ± 1.8	2 ± 1.3	2 ± 0.9	1 ± 0.6
80	8 ± 1.9	13 ± 3.1	13 ± 3.0	14 ± 3.3	11 ± 2.7	10 ± 2.8	7 ± 3.1	4 ± 2.0	3 ± 1.5	2 ± 1.3	1 ± 0.8

Δ*_*J*_’s are given in columns for* Δ*_*I*_’s described in rows. The bold fonts are used to lead the eye to the most probable transitions, occurring after large accelerations* Δ*_*I*_ = − 48 ms and larger decelerations* Δ*_*I*_ = 48 ms, which are distinct from no-change events. These numbers result in the clock-like picture in Figure 4*.

The structure of the plots **A** and **T** is complex. Nevertheless, the gradient plots shown in the second line of Figures 1 and 2 reveal the basic geometrical properties of this structural complexity. Namely, we can observe how the probability changes under the modification of the arguments. The heads of the arrows indicate the direction in which the decrease is the strongest. The arrows’ lengths correspond to the magnitude of this decrease.

In the last rows of Figures 1 and 2, we show graphs of the networks resulting from the signals studied, obtained by Pajek software (Batagelj and Mrvar, 2002). When presenting the networks in their graph form, we applied a special binning which allowed us to represent the crucial effects of aging in the clearest way. In the case of Figure 1, the RR-interval signals were binned at 48 ms. As a consequence, the loop at the vertex 0 of the matrix **A** approximates the probability that two subsequent RR-increments were both smaller than 48 ms. The growth of the width of these loops with age for the groups of older people points to the increasing presence of small changes in the dynamics (we add numbers to these loops to underline this observation). Together with this, the number of transitions greater than 48 ms is reduced.

The size 48 ms of a deceleration or an acceleration is close to the 50 ms value, which is the base of the popular index of vagal activity pNN50, describing the ratio of changes larger than 50 ms in a signal (TaskForce, 1996). Therefore, in the last row of Figure 2, we show parts of **T** networks describing the transition probability conditioned by an acceleration −48 ms and a deceleration + 48 ms. For the graphs of these networks, we used binning of RR-intervals equal to 16 ms. The conditional probabilities for other transitions obtained with this binning are presented in Table 1. The most probable transitions which are conditioned by a ±48 ms change are shown in bold.

**Figure 3:**
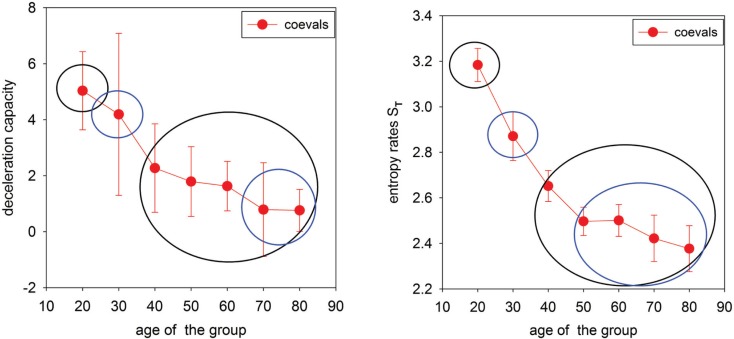
**Summary indices for network representations of age-induced alternations in heart rate dynamics**. **Left**: Deceleration capacity (median ± larger distance to the quartile) calculated for individual *δRR* signals and then pooled in the age groups. Circles separate statistically different groups found by Kruskal–Wallis ANOVA on Ranks with the Dunn method used for comparisons between pairs. **Right**: Transition entropy (mean ± SEM) calculated for *δRR* signals pooled in age groups. Circles separate statistically different groups found by ANOVA with the Holm–Sidak method used for comparisons between pairs.

Finally, in Figure 3, properties of networks **A** and **T** are summarized in plots describing the decay of the deceleration capacity and entropy rate when the age of the group increases.

## 4 Discussion

The arrangement of the contour lines labeled 1% in the contour plots in Figure 1 (which represent pairs of events (δRR_*i*_, δRR_*i*__+1_) occurring with 1% probability) and lines labeled 0.06 in the contour plots in Figure 2 (describing the probability of a transition *δRR_i_*_+1_ given that a transition *δRR_i_* has occurred), together with values collected in Table 1, reveal the dominant change in the heart beat dynamics caused by aging. Namely, the variability of possible (*δRR_i_*, *δRR_i_*_+1_) events becomes gradually reduced with aging, which is further evidenced by the following mechanisms.

First, the reduction in the dynamics implies that the dynamics becomes increasingly dependent on the small *δRR*, namely it involves RR-increments of a size smaller than approximately 32 ms. Network graphs in Figure 1, bottom row, constructed for the RR-signals binned with 48 ms, extract parts of the dynamics focused on such small RR-increments and represent them together as a loop (0,0). We observe that the regime dominated by the small change dynamics covers from about 17 ± 3% at the age of 20, through 34 ± 3% at the age of 40, and 39 ± 2% at the age of 60, to 46 ± 2% at the age of 80. The substantial increase in the lengths of the arrows in the gradient plots in Figure 1, middle row, is also a manifestation of this observation.

Second, there is an important qualitative transition in the probability of observing a pair (δRR_*i*_, δRR_*i*__+1_) when any large *δRR* is encountered, i.e., an acceleration or a deceleration of a size greater than about 40 ms happens. The network graphs in Figure 2, bottom row, together with the values in Table 1, exemplify these changes as follows.

- 20s: both events, a large acceleration and a large deceleration, are slightly more likely to be followed by a deceleration of a size not greater than the preceding RR-increment. Specifically, according to the values presented in Table 1, we observe that:
  – after acceleration *δRR_i_* = −48 ms, the probability of seeing a deceleration of a size not greater than *δRR_i_* = 48, namely *P*(16 ≤ *δRR_i_*_+1_ ≤ 48) is 31 ± 2.9%, and the probability of seeing an acceleration of a similar size, *P*(−48 ≤ *δRR_i_*_+1_ ≤ −16) is 24 ± 2.0%;
  – after deceleration *δRR_i_* = 48 ms, the probability of seeing a deceleration of a size not greater than *δRR_i_* = 48, *P*(16 ≤ *δRR_i_*_+1_ ≤ 48) is 31 ± 2.9%, while the probability of seeing an acceleration of a similar size *P*(−48 ≤ *δRR_i_*_+1_ ≤ −16) is 26 ± 2.0%.
- 40s: despite a noticeable reduction in the overall variability, there is a strong increase in the probability of observing a large deceleration after a large acceleration or large deceleration. Using the description provided for the case of the 20s, we have:
  – after *δRR_i_* = −48 ms: *P*(16 ≤ *δRR_i_*_+1_ ≤ 48) = 40 ± 4.5% and *P*(−48 ≤ *δRR_i_*_+1_ ≤ −16) = 27 ± 2.2%,
  – after *δRR_i_* = 48 ms: *P*(16 ≤ *δRR_i_*_+1_ ≤ 48) = 34 ± 3.5% and *P*(−48 ≤ *δRR_i_*_+1_ ≤ −16) = 29 ± 3.3%.
- 60s: after a large deceleration, although there is still a large probability of seeing a deceleration, there is also a large probability of seeing an acceleration. In particular,
  – after *δRR_i_* = −48 ms: *P*(16 ≤ *δRR_i_*_+1_ ≤ 48) = 48 ± 6.5% and *P*(−48 ≤ *δRR_i_*_+1_ ≤ −16) = 23 ± 3.3%,
  – after *δRR_i_* = 48 ms: *P*(16 ≤ *δRR_i_*_+1_ ≤ 48) = 41 ± 4.8% and *P*(−48 ≤ *δRR_i_*_+1_ ≤ −16) = 35 ± 4.2%.
- 80s: large accelerations and decelerations are likely to occur alternately, which causes RR-intervals to change around the mean value in a pendulum-type motion rather than as a stochastic walk:
  – after *δRR_i_* = −48 ms: *P*(16 ≤ *δRR_i_*_+1_ ≤ 48) = 45 ± 6.9% and *P*(−48 ≤ *δRR_i_*_+1_ ≤ −16) = 23 ± 3.1%,
  – after *δRR_i_* = 48 ms: *P*(16 ≤ *δRR_i_*_+1_ ≤ 48) = 15 ± 4.2% and *P*(−48 ≤ *δRR_i_*_+1_ ≤ −16) = 53 ± 8.5%.

The changes described above can be compared to the transition from equilibrium-like fluctuations in subjects in their forties to pendulum-type dynamical responses in subjects in their eighties. The gradient plots **GA** demonstrate additionally the increase of the steepness of the distributions **A** around the no-change event, and the differences in the symmetry of the events which occur in the elderly.

Since the occurrence of large RR-increments could be related to either strong vagal activation or to vagal withdrawal combined with sympathetic stimulation (Martini et al., 2011; Poirier, 2014), we can considered our results to reveal diminishing vagal activity and/or diminishing cooperation between the vagal and the sympathetic part of the ANS with aging. This observation is supported directly by a strong decay in the deceleration capacity; see Figure 3 left.

The mean transition matrices shown in Figure 2 illustrate the short-term dependence between the subsequent heart beats. We see that the core probability, which involves small variations around the no-change event at the age of 20, spreads to events other than the no-change event when the subjects are in their forties. Moreover, the probability of the next *δRR*(*i* + 1) appears to be independent of the last *δRR*(*i*) for changes from −32 ms to 32 ms. This independence spreads, but for the decelerations only, up to 64 ms in the case of subjects in their sixties. Accelerations are characterized by the probabilities dependent on the size of the preceding acceleration. The greater the acceleration, the larger the probability is of a deceleration in the next step. At the age of 80, such alternations, which can be compared to pendulum dynamics, are also observed in the decelerations.

This pendulum-like dynamics is damped because the increment which follows a given event is smaller. Moreover, such gradual stabilization of the transition probabilities with age is evidenced by a decrease in the entropy rate, see Figure 3. Because of this effect, the location of the maxima in the transition probability depends on Δ*_J_*, which is clearly demonstrated in the gradient plots **GT**. The occurrence of the pendulum-like dynamics is represented in detail in Table 1, where the results obtained from signals binned with *bin* = 16 ms are provided. From Table 1 we see that, although transitions to no-change events are the most probable in elderly humans, independently of the size of RR-increments, the pendulum oscillations are present.

**Figure 4:**
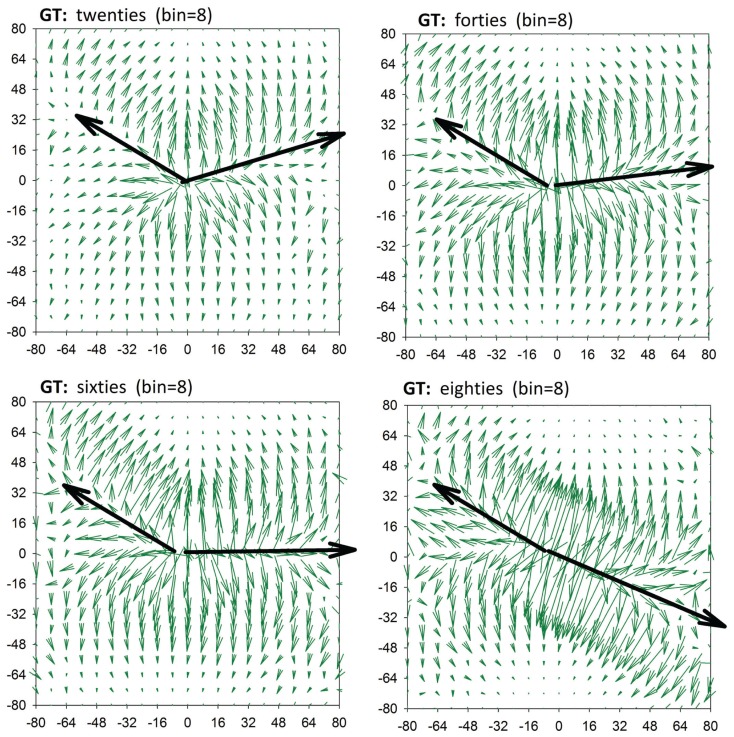
A clock of autonomic aging made by linking points for which values of T(I,*) are maximal.

RR-signals are known to have 1/*f* scaling properties with the Hurst exponent gradually approaching *H* = 0.2 with aging (Struzik et al., 2006) for all but the shortest timescales (>20 heart beats). This phenomenon corresponds with increasingly antipersistent behavior of RR-signals at the smallest temporal resolutions with increasing age. Such simple antipersistent dynamics is also characteristic of the heart rhythm of patients after heart transplantation, that is, for subjects with denervated hearts (Makowiec et al., 2014). Table 1 supports the conclusion that the antipersistent features are attributed to accelerations independently of the subject’s age, while the decelerations change from a slight persistency to antipersistency with the subject’s age. We can picture the changes by drawing lines in **GT** plots, which connect maxima of transition probabilities, see Figure 4. These lines metaphorically resemble the hands of a clock. We can indeed read the passing of time in our “chronograph” of heart rate dynamics: 10 min past ten for subjects in their twenties, a quarter past ten for subjects in their sixties, and 20 past ten for subjects in their eighties. The relative angle between the arrows – the hands of the metaphoric chronograph – reflects the degree of antipersistency; however, the absolute position of the arrows, the ridges of the maxima of the gradient, is also of physiological relevance.

The increase in pendulum-like oscillatory antipersistency may involve the very mechanism responsible for the decrease in entropy with age, also observed previously (Viola et al., 2011; Makowiec et al., 2015b). It is as of yet unclear whether solely ANS neural factors are behind the physiological origins of this mechanism. It may be related to feedback compensatory dynamics due to the decrease in baroreflex sensitivity with age. Possibly, the gradual stiffening of the vasculature and hypertrophy of the cardiac muscle with age plays a role in the genesis of this effect. Both these factors may be present and may convolve with the altering spectrum of autonomic control. The progressive impairment of the autonomic balance with age (Crasset et al., 2001; Brandenberger et al., 2003) does not resolve the question of the direction of the causal relationship – whether the increase in the sympathetic dominance is responsible for the diminishing appearance of deep sleep or whether the lack of deep sleep in the elderly leads to lesser evidence of the vagal tone. The debate remains open, as some authors argue (Schmitt et al., 2009) that the structure of sleep dominates over aging processes and “overrides” the effects of aging on HRV. This could potentially explain the fact of decreased vagal presence despite the progressive loss of sympathetic neurons with age (Struzik et al., 2006).

Sleep can in general be assumed to be a period of human activity which is free of external stimulation. Therefore, the nocturnal part of a 24-h Holter recording provides a good possibility of observing the state of the autonomic baseline (Stein and Pu, 2012; Tobaldini et al., 2013; Chouchou and Desseilles, 2014). Indeed, a recent review (Stein and Pu, 2012) advocates using nocturnal records for HRV analysis, while (Chouchou and Desseilles, 2014) reviews tantalizing possibilities of the quantitative assessment of not only autonomic but also higher nervous centers through analysis of sleep HRV records. However, as sleep is organized in cycles, each lasting about 90 min, stages of slow wave sleep (non-rapid eye movement sleep: NREM) are followed by rapid eye movement sleep (REM) (Guyton and Hall, 2006). HRV has indeed been found to be strongly affected by the sleep organization – vagal modulation and sympathetic activity follow the sleep stages (Monti et al., 2002; Schumann et al., 2010). It is generally recognized that NREM is characterized by a predominant parasympathetic drive, while the REM stage exhibits increased sympathetic modulation and a loss of parasympathetic control. Also, the brain is more active in REM sleep. REM sleep and arousal from sleep presumably reflect central autonomic commands leading to transient periods of tachycardia (Trinder, 2008). It has been found that these transitions lead to significantly higher values of SD of RR intervals for REM sleep than for deep NREM sleep, independently of age (Schmitt et al., 2009). However, the index of very short-term variability, the square root of the mean of the sum of the squares of differences (RMSSD) has been found to be insensitive to non-stationarities caused by bursts of sympathetic activity during different sleep stages independently of age (Schmitt et al., 2009). Since our investigations are related to short-term variability, we can suppose that our results are not strongly influenced by sleep stages or sleep transitions.

## 5 Summary

The complexity of the mechanisms involved in the phenomenon of heart rate variability is challenging, and the existing measures of this complexity are not satisfactory. The proposed method provides easily readable graphs revealing the complexity of the autonomic control, to date not assessed in comparable detail. The intricate topological structure of these graphs helps to reveal the effects of sympathetic and vagal modulation on heart rate dynamics. Additionally, the two measures proposed provide the means to summarize the graphs’ structure: (i) the approximate deceleration capacity and (ii) the entropy rates.

Significant age-related changes in heart beat-to-beat dynamics have been observed using the geometrical tools of the method introduced: from the large variety of possible movements at the age of 20, to the strong antipersistency similar to pendulum dynamics, which becomes dominant in subjects in their eighties.

Aging and its effects on the entire cardiovascular system, and specifically on the autonomic regulation, are themselves complex processes and capturing this in HRV remains an open problem. In particular, causal, possibly non-linear interactions (Porta et al., 2014) may be involved. Our geometrically oriented methodology may contribute to the range of analytic methods and help in further elucidating complex mechanisms of aging.

## Author Contributions

DW, AK, MZB: the acquisition of data for the work; DM: drafting of the work; DM, ZRS: critically revising the significant intellectual content of the work, and final approval of the published version.

## Conflict of Interest Statement

The authors declare that the research was conducted in the absence of any commercial or financial relationships that could be construed as a potential conflict of interest.
